# Citrus Changshan Huyou extracts as natural GLP-1R agonists attenuate atherosclerosis via the GLP-1R/PKA/CREB pathway: a functional food perspective

**DOI:** 10.3389/fnut.2026.1899085

**Published:** 2026-08-05

**Authors:** Yue-Yue Fang, Chun-Yue Bao, Wan-Zhao Liu, Peng-Fei Xie, Chao-Ran Wang, Xiao-Lin Zhang, Jian-Mei Li

**Affiliations:** 1 School of Food Science and Pharmaceutical Engineering, Nanjing Normal University, Nanjing, China; 2 State Key Laboratory of Phytochemistry and Natural Medicines, Dalian Institute of Chemical Physics, Chinese Academy of Sciences, Dalian, China

**Keywords:** atherosclerosis, Citrus Changshan Huyou extracts, functional food ingredient, GLP-1R/ PKA/CREB signaling pathway, glucagon-like peptide 1 receptor

## Abstract

**Introduction:**

Atherosclerosis (AS)-triggered cardiovascular diseases constitute a major global public health crisis. The pathological progression of AS is characterized by abnormal lipid accumulation, inflammatory cell infiltration and atherosclerotic plaque buildup in arterial walls. Glucagon-like peptide-1 receptor agonists (GLP-1RAs) are well known for hypoglycemic effects, and accumulating evidence proves they exert extra cardiovascular protective effects. This research aimed to explore whether Citrus Changshan Huyou extracts (HY) function as natural food-origin GLP-1RAs and further verify their anti-atherosclerotic activity and underlying mechanisms.

**Methods:**

A GLP-1R-targeted cell screening model was adopted to detect the GLP-1 agonistic activity and cytotoxicity of HY *in vitro*. The five major flavonoid monomers of HY (eriocitrin, neoeriocitrin, narirutin, naringin and neohesperidin) were isolated and individually validated for GLP-1R activating capacity at the cellular level. A high-fat diet-induced ApoE^−^/^−^ mouse model was established to assess the *in vivo* efficacy and biosafety of HY. Subsequent mechanistic experiments were conducted to clarify the regulatory role of HY on the intestinal GLP-1R/PKA/CREB signaling cascade and downstream vascular aging and inflammatory responses.

**Results:**

Cellular assays confirmed that HY possessed prominent GLP-1R agonistic activity with negligible cytotoxicity, and all five intrinsic flavonoid components of HY independently activated GLP-1R. In animal experiments, HY intervention markedly relieved excessive weight gain, corrected disordered blood lipid levels and shrank atherosclerotic plaque lesions in the aorta, without any detectable toxic reactions. Mechanistically, HY activated intestinal GLP-1R/PKA/CREB signaling, which further suppressed the expression of vascular senescence-associated proteins and alleviated inflammatory infiltration in aortic tissues.

**Discussion:**

This study verifies that HY is a safe and potent natural GLP-1RA derived from edible citrus raw materials with robust anti-atherosclerotic performance. The intestinal GLP-1R/PKA/CREB pathway mediates the dual protective effects of HY against vascular senescence and aortic inflammation. Our findings provide solid experimental support for the development of novel cardiovascular-protective functional food raw materials based on Changshan Huyou extracts.

## Graphical abstract

**Figure:**
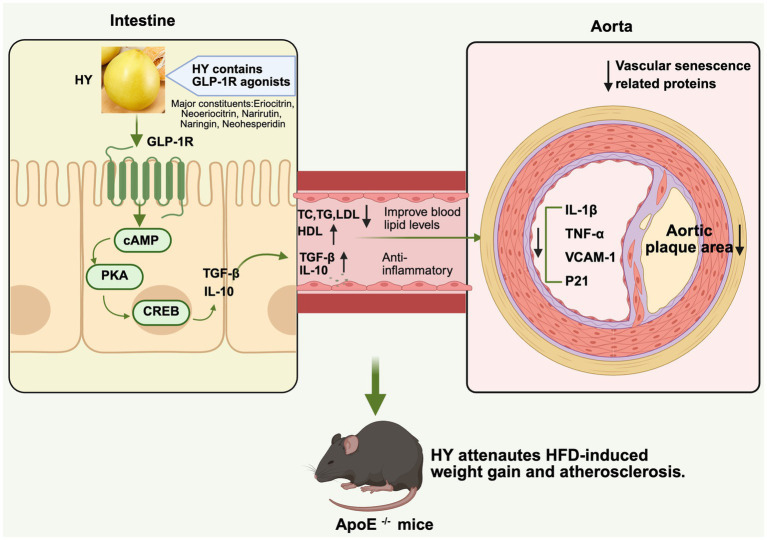

## 1 Introduction

GLP-1 receptor agonists (GLP-1RAs) are structural analogs of the endogenous hormone glucagon-like peptide-1 (GLP-1). They primarily mediate and exert multiple physiological regulatory effects by activating GLP-1 receptors (GLP-1Rs), which are widely distributed in various tissues, including the pancreas, intestines, and cardiovascular system (1). As a G protein-coupled receptor (GPCR), glucagon-like peptide-1 receptor (GLP-1R) elevates the cellular level of cyclic adenosine monophosphate (cAMP) upon activation to activate protein kinase A into phosphorylated P-PKA, which further mediates the phosphorylation of cAMP response element-binding protein (CREB) and ultimately regulates the transcription of a series of downstream target genes (2–4). The GLP-1R/PKA/CREB signaling pathway serves as a key molecular hub through which GLP-1 receptor agonists exert their wide-ranging physiological regulatory and pharmacological effects (5). Previous studies have demonstrated that natural bioactive compounds and traditional Chinese herbal formulas can also intervene in carbohydrate and lipid metabolism disorders by regulating this pathway. For example, *Achyranthes bidentata* polysaccharide ameliorates type 2 diabetes mellitus by gut microbiota-derived short-chain fatty acids-induced activation of the GLP-1/GLP-1R/PKA/CREB/INS pathway (6); Zexie Decoction can regulate the browning of white adipose tissue and the activation of brown adipose tissue through the GLP-1R/PKA/CREB pathway (7).

In recent years, research has demonstrated that activation of GLP-1R produces multifaceted cardiovascular protective effects independent of its hypoglycemic action, specifically including improved vascular endothelial function, inhibition of atherosclerotic plaque formation, modulation of the body’s inflammatory response, alleviation of oxidative stress damage, and improved myocardial energy metabolism (8, 9). Currently, most GLP-1RAs in clinical use are peptide-based formulations, which generally have limitations such as the need for injection, high development and usage costs, and a tendency to cause gastrointestinal adverse reactions (10–12). Unlike injectable peptide-based GLP-1RAs with high costs and gastrointestinal side effects, food-derived GLP-1RAs offer oral bioavailability, safety, and low cost. They can be incorporated into daily diets as supplements, enabling long-term adherence and preventive use, highlighting the value of screening natural foods for safe, affordable cardiovascular strategies (13). As research continues to advance, a variety of structurally diverse non-peptide GLP-1RAs have been reported (14–16). Consequently, the scope of GLP-1R agonist research has gradually expanded from chemically synthesized small-molecule drugs to the field of naturally active substances. Mounting preclinical and clinical evidence has confirmed that plant-derived natural antioxidants possess great potential against metabolic syndrome and atherosclerotic cardiovascular complications, which further supports the development of food-based GLP-1R agonists for cardiometabolic intervention (17, 18).

The ongoing paradigm shift from traditional medicine-food homology to modern, evidence-based food-medicine homology underscores the therapeutic potential of characteristic economic crops (19). Citrus Changshan Huyou (HY) is a natural hybrid of lime and pomelo endemic to China and has been recognized as a characteristic economic crop (20). Accumulating evidence has demonstrated that HY and its bioactive constituents possess a range of biological activities, including antioxidant effects via scavenging free radicals and reducing oxidative stress markers, as well as anti-inflammatory properties through downregulation of pro-inflammatory cytokines such as interleukin-6 (IL-6) and tumor necrosis factor-*α* (TNF-α) (21–23). Additionally, several studies have shown that citrus flavonoids, including those present in HY, can effectively regulate lipid metabolism disorders and attenuate obesity-related metabolic disturbances (24–26). Reportedly, extracts from unripe citrus fruits regulate the expression of key genes involved in lipid metabolism and adipogenesis by activating the adenosine monophosphate-activated protein kinase (AMPK) signaling pathway while simultaneously inhibiting the mitogen-activated protein kinase (MAPK) signaling pathway, thereby suppressing adipocyte differentiation and lipid accumulation. As such, they hold potential for development as natural ingredients with fat-reducing properties (27). Similar research has shown that naringin alleviates atherosclerosis in ApoE^−^/^−^ mice by modulating cholesterol metabolism, which contributes to the remodeling of the gut microbiota (28). Despite these promising findings, no study to date has investigated whether HY or its major components can activate the GLP-1 receptor, nor has the potential cardiovascular protective effect mediated by GLP-1R activation been explored. This knowledge gap limits the understanding of HY’s full pharmacological potential and its possible application in functional foods targeting cardiometabolic health.

This study seeks to identify novel GLP-1RAs from HY and to delineate their mechanisms of action against atherosclerosis in a high-fat diet-fed ApoE^−^/^−^ mice model.

## 2 Materials and methods

### 2.1 Materials and chemicals

#### 2.1.1 HY samples

Dried HY peels (5 kg) were subjected to extraction with 60 L of 70% (v/v) aqueous ethanol by maceration for 20 h. This extraction process was repeated twice with fresh solvent. The combined extracts were filtered through filter paper and concentrated under reduced pressure to remove ethanol. The resulting concentrated aqueous residue was diluted with water, followed by suction filtration. Then the filtrate was loaded onto a D101 macroporous adsorption resin column. The column was sequentially washed with 3 column volumes each of purified water and 20% aqueous ethanol to eliminate impurities. The target compounds were subsequently eluted with 50% aqueous ethanol. The collected eluate was concentrated under reduced pressure and finally dried by spray drying to obtain the enriched HY as a powder. The enriched fraction of HY was separated by preparative high-performance liquid chromatography (HPLC) with fraction collection, using a Unitary C18 column (4.6 mm × 250 mm, 5 μm).

**Figure 1:**
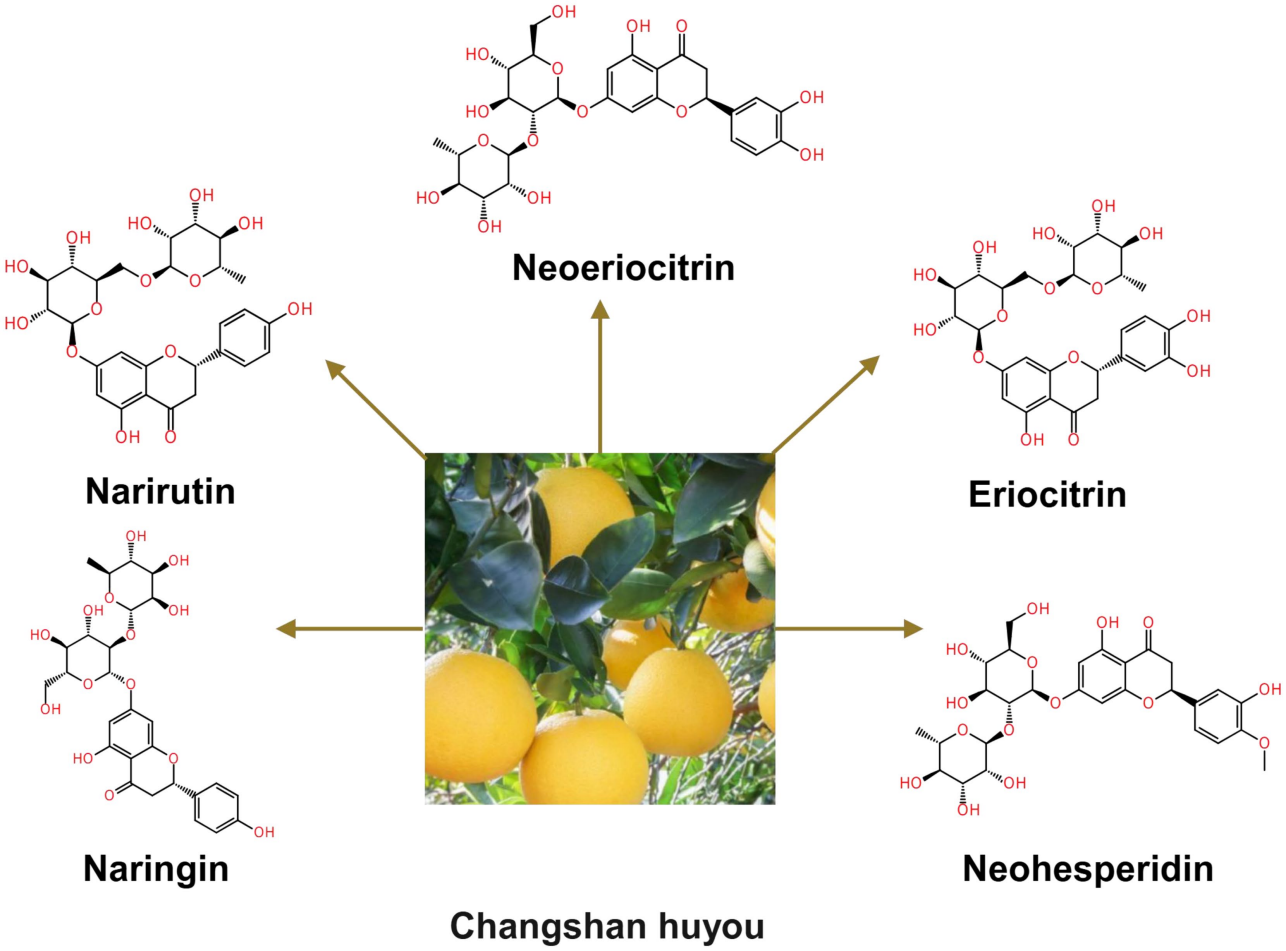
Chemical structure of active ingredients in HY.

224.36 grams of HY peel extract were obtained from 5 kilograms of dried HY peels. The chromatograms are shown in Supplementary Figure S1. These five components were structurally identified, and their mass spectra (MS) are shown in Supplementary Figure S2. All spectral data were consistent with the reference standard values reported in the literature, confirming that these five compounds are Eriocitrin, Neoeriocitrin, Narirutin, Naringin, and Neohesperidin. Quantitative analysis demonstrated that the five characteristic flavonoids, namely Eriocitrin, Neoeriocitrin, Narirutin, Naringin, and Neohesperidin, accounted for 3, 20, 3, 47, and 25% of the total mass of Citrus Changshan Huyou peel extract, respectively. The chromatographic purity of all five flavonoid monomers exceeded 98%, and their HPLC chromatograms are shown in Supplementary Figure S3. The chemical structures of the active ingredients in HY are shown in Figure 1.

#### 2.1.2 Chemicals and reagents

GLP-1R lentivirus was purchased from General Biology Co., Ltd. (Anhui, China). cAMP ELISA Kit and Exendin-9 (9–37) were purchased from Sangon Biotech (Shanghai) Co., Ltd. (Shanghai, China). RIPA whole protein extraction kit, BCA protein concentration determination kit, SDS-PAGE protein loading buffer, and SDS-PAGE gel preparation kit were purchased from Beijing Labgic Technology Co., Ltd. (Beijing, China). Commercially available assay kits of total cholesterol (TC), triglycerides (TG), high-density lipoprotein-cholesterol (HDL-C), low-density lipoprotein-cholesterol (LDL-C) were obtained from Nanjing Jiancheng Bio-Engineering Institute Co., Ltd. (Nanjing, China). DMEM was purchased from Gibco (CA, U.S.A.). Fetal bovine serum, penicillin–streptomycin and pancreatin with 0.25% EDTA were purchased from Nanjing SenBeiJia Biological Technology Co., Ltd. (Nanjing, China). The Cell Counting Kit-8 was derived from Beyotime Biotechnology (Shanghai, China).

The antibodies applied in our work were as following: anti-P21CDKN1A (1:1000, sc-6246, Santa Cruz, mouse), anti-TGFβ1 (1:1000, WL02998, Wanleibio, rabbit), anti-VCAM1 (1:1000, WL02474, Wanleibio, rabbit), anti-IL-1 (1:5000, ABS830030, ABSIN, mouse), anti-GLP-1R (1:1000, sc-6246, Santa Cruz, mouse), anti-PKA (1:1000, WL02998, Wanleibio, rabbit), anti-CREB (1:1000, WL02474, Wanleibio, rabbit), anti-p-CREB (1:1000, WL02474, Wanleibio, rabbit), anti-GAPDH (1:5000, ABS830030, ABSIN, mouse), anti-β-actin (1:5000, #T0022, Affinit, mouse).

### 2.2 Cell culture

Human kidney 293 (HEK293) cells and STC-1 cells were purchased from National Collection of Authenticated Cell Cultures and cultured in DMEM supplemented with 10% fetal bovine serum (FBS) and 1% penicillin–streptomycin under standard conditions (37 °C, 5% CO2, and 95% humidity).

### 2.3 Cell viability analysis

Cell survival was assessed using a CCK-8 kit. HEK-293 T cells and GLP-1R-HEK-293 T cells were seeded in 96-well plates at a density of 1 × 10^4^ cells per well and incubated for 24 h. Then, cells were treated with 100 μL of HY at varying concentrations (25, 50, 100, 200, and 400 μg/mL) for 24 h. A cell-only group without the sample was included as a blank control, and a medium-only group without cells was used as a zero-adjustment set. Next, 1/10 volume of CCK-8 working solution (CCK-8 and culture medium 1:10 v/v) was added to each well. After 2 h of incubation at 37 °C, the optical density (OD) value at 450 nm was measured using a microplate reader.

### 2.4 GFP-GLP-1R lentivirus transduction

Logarithmically growing HEK-293 T cells were collected and prepared as a single-cell suspension in culture medium supplemented with 10% FBS. The culture medium in each well was replaced with 2 mL of fresh medium containing 5 μg/mL polybrene. Subsequently, 20 μL of the lentiviral supernatant was added to each well. After incubation at 37 °C for 24 h, the medium was replaced again with fresh culture medium without polybrene or virus. At 72 h post-infection, fluorescence was observed under a microscope to assess infection efficiency. Upon confirmation of successful transduction, the medium was replaced with fresh medium containing 2 μg/mL puromycin for selection. The selection medium was refreshed every 3 to 5 days based on its color and the condition of the cells. When the puromycin-resistant cells reached approximately 80–90% confluence, they were expanded for subsequent experiments.

### 2.5 Western blots

Aortic and intestinal tissues were harvested, homogenized in ice-cold lysis buffer (containing 1% PMSF and 1% phosphatase inhibitors), and centrifuged at 12,000 rpm for 15 min at 4 °C to obtain lysates. Protein concentrations were detected using a BCA assay kit with BSA as a standard. Lysates were mixed with one-third volume of loading buffer, heated at 100 °C for 10 min to denature proteins, and separated by SDS-PAGE. Proteins were then transferred onto a PVDF membranes (IPVH00010, Millipore), blocked with 5% skimmed milk, and incubated overnight at 4 °C with a primary antibody, followed by 1-h incubation with secondary antibodies at room temperature. Protein bonds were visualized using the Tanon 5,200 chemiluminescence detection system and band intensities were quantified using Image J software. For sequential detection of different proteins on the same membrane, the membrane was first incubated with the internal reference antibody and visualized. Thereafter, the membrane was stripped using a mild stripping buffer at room temperature for 15–30 min to completely remove the bound primary and secondary antibodies against the loading control. The membrane was then washed thoroughly with TBST, re-blocked with 5% skimmed milk, and re-probed with primary antibodies against other target proteins of interest, followed by secondary antibody incubation and visualization as described above. Using this stripping-and-reprobing method, both the loading control and additional target bands were detected from a single membrane.

### 2.6 Enzyme-linked immunosorbent assay (ELISA)

Levels of cAMP and GLP-1 were measured in cell supernatant by the commercially available ELISA kits.

### 2.7 Molecular docking

The 3D structures of the five active ingredients were retrieved from PubChem,^1^ while GLP-1R structures were obtained from RCSB PDB.^2^ Structural preprocessing was conducted using OpenBabel 2.3.2, PyMol 3.0, and AutoDockTools 1.5.6. Resulting ligand-receptor interactions were visualized through PyMol 3.0 and Discovery Studio.

### 2.8 Animal experiment

Male C57BL/6 J mice (6–8 weeks; 18–22 g) were purchased from GemPharmatech Co., Ltd. (Jiangsu, China). The ApoE^−/−^ mice (6–8 weeks; 18–22 g) colony was bred by our research group. All mice were housed in a specific-pathogen-free (SPF) facility at Animal Research Center, School of Life Sciences, Nanjing Normal University, under controlled conditions (temperature: 22 ± 2 °C; humidity: 40–60%; 12/12-h light/dark cycle) with ad libitum access to food and water and weekly bedding changes. All animal experiments were conducted in accordance with the guidelines outlined in the “Guide for the Care and Use of Laboratory Animals” and were approved by the Animal Ethics Committee of Nanjing Normal University.

Eight male C57BL/6 mice were designated as the WT group. Forty male ApoE^−/−^ mice were randomly and equally divided into five groups (n = 8 per group): a Control group (ApoE^−/−^), a Model group (ApoE^−/−^ + HFD), a Low-dose HY group (ApoE^−/−^ + HFD + 50 mg/kg/day), a Medium-dose HY group (ApoE^−/−^ + HFD + 100 mg/kg/day), and a High-dose HY group (ApoE^−/−^ + HFD + 200 mg/kg/day). Mice in the WT and Control groups were fed a standard chow diet, while those in the HFD model and treatment groups received a high-fat diet. Numerous studies have confirmed that continuous HFD feeding for 6 weeks can induce obvious pathological characteristics in experimental animals of the strain used in this study, and the established animal model exhibits good stability. Throughout the experimental period, HFD induction was established for 6 weeks, followed by an 8-week oral gavage treatment with HY under HFD feeding. The WT group and the Control group received an equal volume of physiological saline via gavage.

Body weight was recorded weekly. At the study endpoint, mice were fasted overnight, euthanized, and samples were collected. Serum and aortic tissues were snap-frozen and stored at −80 °C, while aortic and cardiac tissues for morphological analysis were fixed in 4% paraformaldehyde.

### 2.9 Glucose tolerance test (GTT) and insulin tolerance test (ITT)

GTT was performed by injecting 2 g/kg glucose intraperitoneally after 16-h fasting from 6:00 p.m. to 10:00 a.m. the following day. ITT was performed via intraperitoneal injection of 0.3 U/kg insulin after 6-h fasting from 9:00 a.m. to 3:00 p.m. Blood glucose levels were measured using tail vein blood with Ultra Easy® glucose monitor at 0, 15, 30, 60, 90 and 120 min after insulin injection. The areas under the curve (AUC) were calculated by GraphPad Prism 10 software.

### 2.10 Atherosclerotic, histological, and biochemical analyses

The heart and entire aorta were carefully dissected from the mice to fully expose the vessels. Histological staining of aortic tissues, liver tissues, and white adipose tissue (WAT), including hematoxylin and eosin (H&E) for morphology and Oil Red O (ORO) for lipid accumulation, were performed by Servicebio Co., Ltd. The stained sections were examined with a light microscope (NIB620, Nexcope, China). Quantitative analysis was conducted in a blinded manner using Image J software.

Serum concentrations of TC, TG, LDL-C, and HDL-C were quantified using commercial assay kits, following the manufacturers’ protocols. Absorbance was measured at the specified wavelengths on a microplate reader (FlexA-200, Allsheng, China).

### 2.11 Statistical analysis

All data were reported as mean ± SEM (standard error of mean, indicated by error bars). One-way analysis of variance (ANOVA) followed by Tukey’s test was carried out in SPSS Statistics Software (IBM Corp., Armonk, NY, USA) for statistical analysis between different groups, and *p* < 0.05 was taken to indicate statistical significance. All figures were conducted using GraphPad Prism version 10 software.

## 3 Results

### 3.1 Obtaining stable and highly expressed GLP-1R cell lines

**Figure 2:**
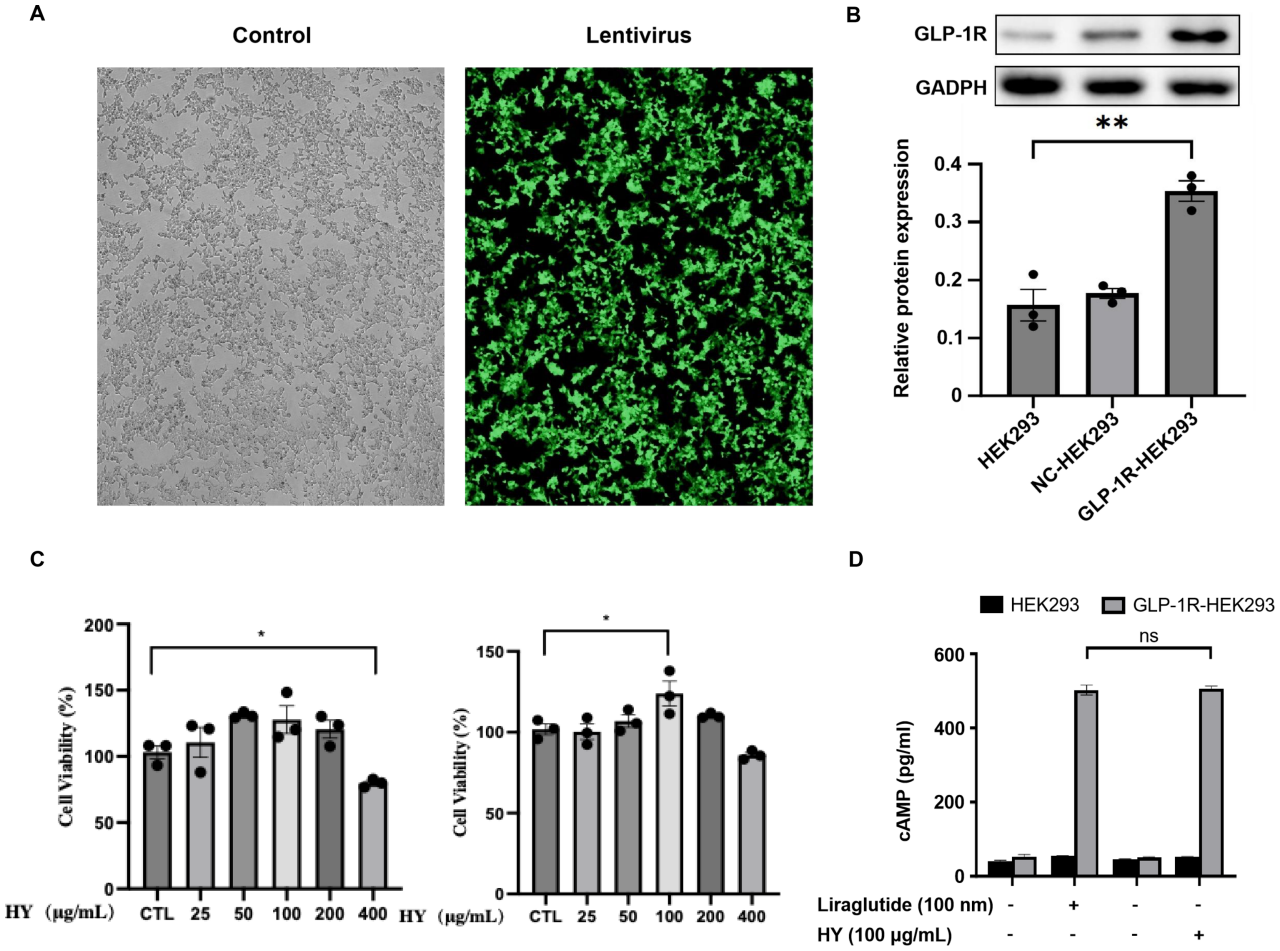
Process of obtaining stable and highly expressed GLP-1R cell lines. **(A)** Fluorescence observation of HEK293 cells at 72 h after lentiviral transfection. **(B)** The GLP-1R protein bands and the quantified relative GLP-1R protein expression levels were determined by the Western blotting assay. **(C)** Effects of different concentrations of HY on the viability of HEK293 and GLP-1R-HEK293 cells. **(D)** Levels of intracellular cAMP in HEK293 and GLP-1R-HEK293 cells following stimulation with HY or Liraglutide. Data are presented as mean ± SEM, (*n* ≥ 3), ns, not statistically significant, *p < 0.05, **p < 0.01.

To screen bioactive compounds from natural products, we established a cell line with high GLP-1R expression. HEK293 cells were infected with lentivirus for 72 h, and the results after infection are shown in Figure 2A. The protein expression of GLP-1R was determined in blank HEK293 cells, NC-HEK293 cells, and GLP-1R-HEK293 cells. Compared with blank HEK293 and NC-HEK293 cells, the expression of the targeted GLP-1R protein was significantly upregulated in GLP-1R-HEK293 cells (Figure 2B). Thus, a stable cell line with high GLP-1R expression was successfully established. Next, HEK293 and GLP-1R-HEK293 cells were treated with HY at concentrations of 25, 50, 100, 200, and 400 μg/mL. Cell viability was assessed using the CCK-8 assay. The left image shows the effect of HY on the cell viability of HEK293 cells, and the right image shows the effect of HY on the cell viability of GLP-1R-HEK293 cells. As shown in Figure 2C, HY with concentrations ranging from 25 to 200 μg/mL did not affect the survival rate of either cell line. Given this, the cells were treated with vehicle control, 100 nM liraglutide, or 100 μg/mL HY. Following 4–6 h of incubation, the culture medium was discarded. A specified volume of cell lysate was then collected, and intracellular cAMP concentration was measured. The results demonstrated that stimulation with HY or liraglutide significantly increased cAMP levels in GLP-1R-HEK293 cells (Figure 2D). These findings provide preliminary evidence that HY act as GLP-1RAs.

### 3.2 Validating the specific binding of HY and active ingredients to GLP-1R

**Figure 3:**
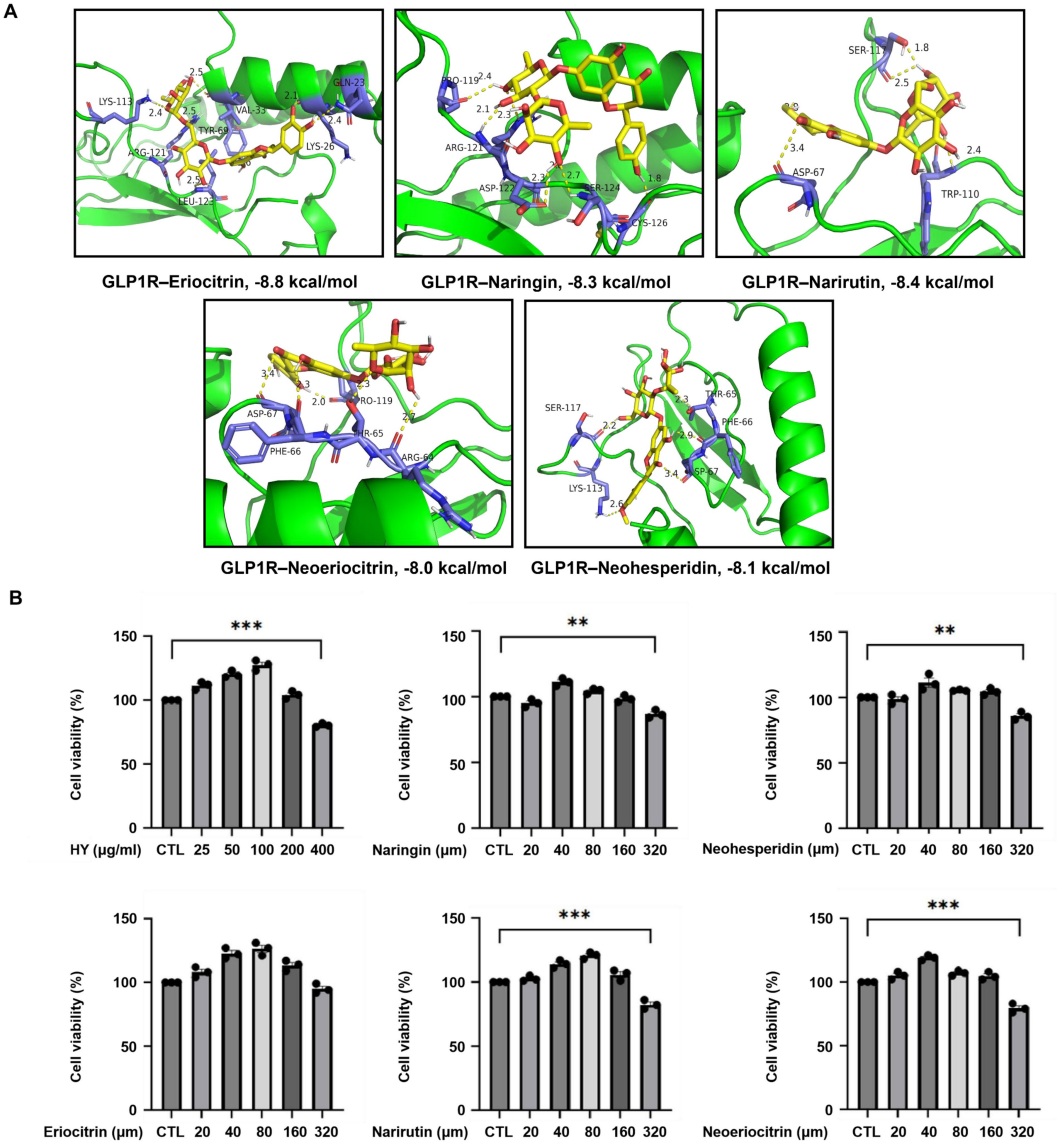
Preliminary binding mechanism and cell viability. **(A)** Molecular docking of the crystal structures of the five active constituents with the 3D model of the GLP-1R protein. **(B)** Effects of the five active constituents on the viability of STC-1 cells. Data are presented as mean ± SEM, (*n* ≥ 3), *p < 0.05, **p < 0.01, ***p < 0.001 VS CTL.

To investigate the potential mechanism by which the active constituents of HY effect, we performed molecular docking analysis to evaluate the binding affinity and interaction mode of these five compounds with GLP-1R. The results show that their calculated minimum binding energies were −8.3, −8.1, −8.8, −8.4, and −8.0 kcal/mol (Figure 3A). The simulation results indicated that all five compounds could specifically bind to GLP-1R, suggesting their potential as direct receptor agonists.

**Figure 4:**
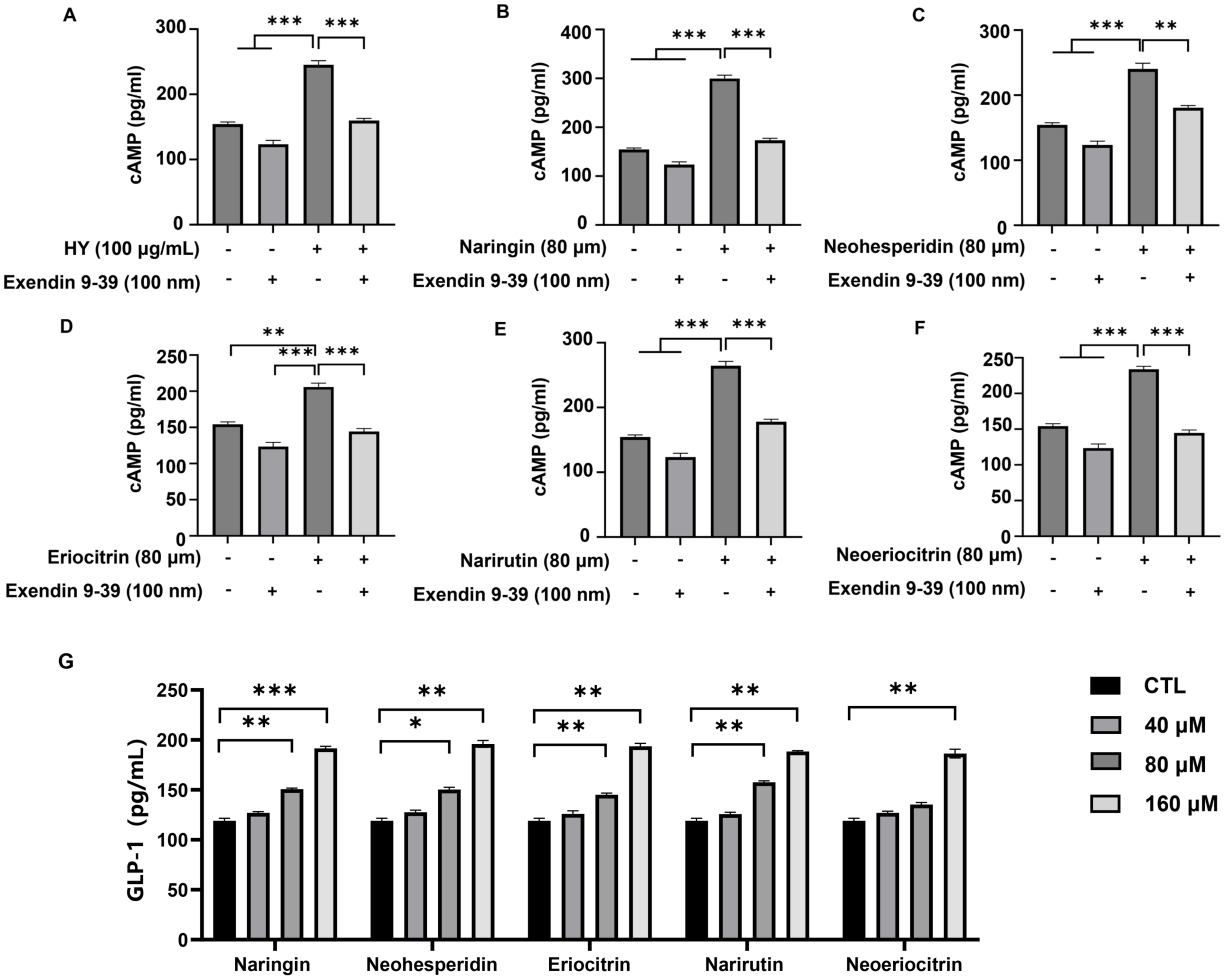
Activation effect of HY and active components on GLP-1R in STC-1 cells. **(A)** Changes in cAMP production of STC-1 cells stimulated by HY. **(B)** Changes in cAMP production of STC-1 cells stimulated by Naringin. **(C)** Changes in cAMP production of STC-1 cells stimulated by Neohesperidin. **(D)** Changes in cAMP production of STC-1 cells stimulated by Eriocitrin. **(E)** Changes in cAMP production of STC-1 cells stimulated by Narirutin. **(F)** Changes in cAMP production of STC-1 cells stimulated by Neoeriocitrin. **(G)** Determination of GLP-1 levels in cell culture supernatant. Data are presented as mean ± SEM, (*n* ≥ 3), ** *p* < 0.01, *** *p* < 0.001.

Subsequently, this computational prediction was validated in STC-1 cells. Cells were treated with 0.1% DMSO or five active components (at concentrations of 20, 40, 80, and 160 μM) for 24 h, followed by CCK-8 assay. These five components exhibited no cytotoxicity at these concentrations (Figure 3B). Treatment with the HY and five active compounds significantly increased intracellular cAMP levels in a dose-dependent manner, and this increase was effectively inhibited by pre-treatment with Exendin (9–37), a specific and competitive antagonist of GLP-1R (Figures 4A–F). This blocking effect confirms that HY increase intracellular cAMP levels primarily by specifically activating GLP-1R, rather than through non-specific or off-target pathways.

Furthermore, to investigate another indirect mechanism of receptor activation, the effects of these five components on the secretion of endogenous GLP-1 were examined. STC-1 cells were treated with five constituents at concentrations of 40, 80, and 160 μM for 24 h, respectively. The results showed that several of these compounds could upregulate the secreted GLP-1 level in a concentration-dependent manner (Figure 4G). This finding suggests that, besides directly binding to the receptor, the active constituents of HY can also promote GLP-1R pathway engagement by stimulating the secretion of endogenous GLP-1, thereby triggering a positive feedback loop.

### 3.3 HY attenuated HFD-induced obesity, dyslipidemia, and glucose intolerance in ApoE^−^/^−^ mice

**Figure 5:**
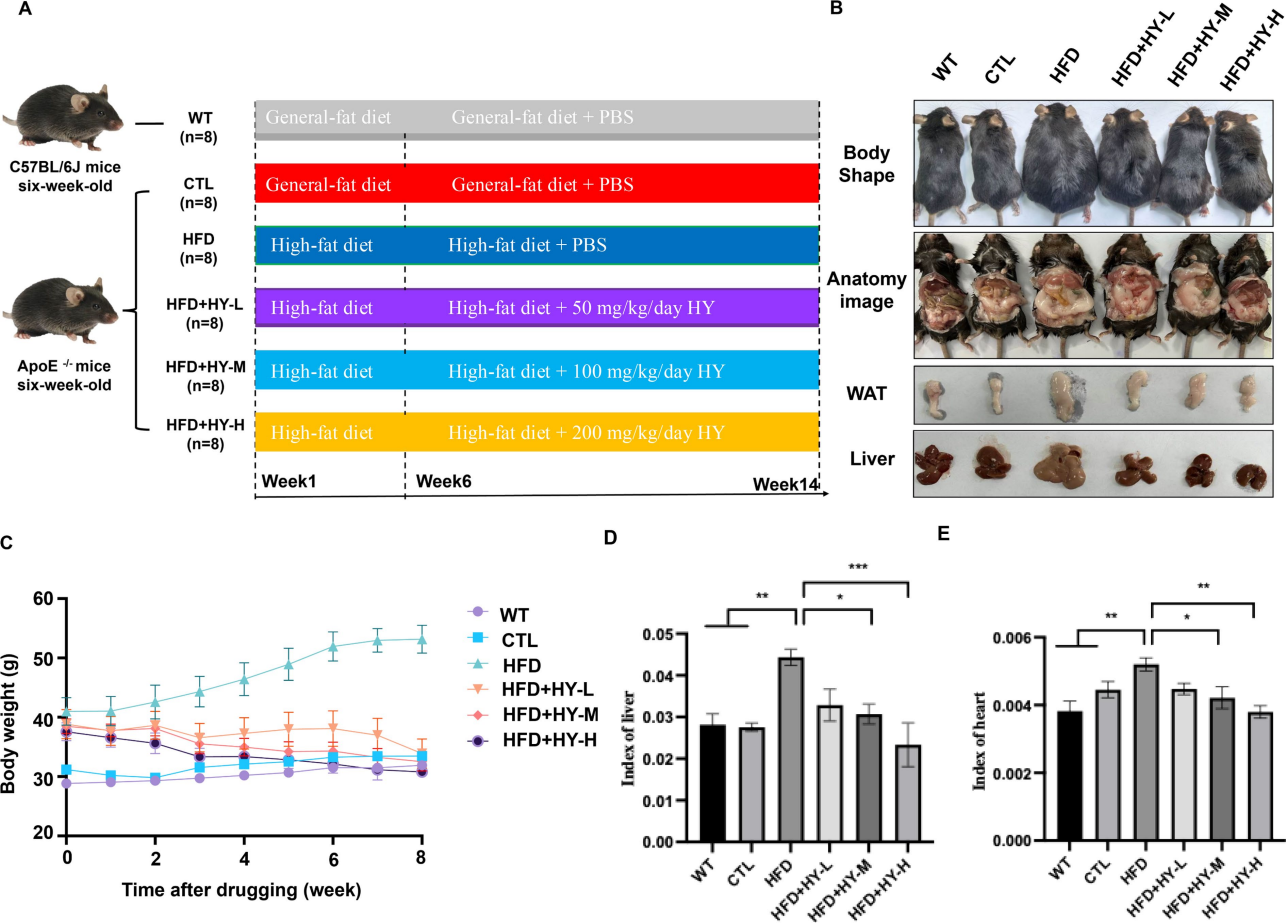
Effect of HY on body weight and organ coefficient in ApoE^−^/^−^ mice. **(A)** Schematic diagram of the experimental design for administering HY to mice. **(B)** Representative photos of body shape, WAT and liver. **(C)** Dynamic changes in weight. **(D)** Mouse liver coefficient. **(E)** Mouse heart coefficient. WT, Wild-type mice; CTL, ApoE−/−; HFD, ApoE−/− + HFD; HFD + HY-L, ApoE−/− + HFD + 50 mg/kg/day HY; HFD + HY-M, ApoE−/− + HFD + 100 mg/kg/day HY; HFD + HY-H, ApoE−/− + HFD + 200 mg/kg/day HY. Data are presented as mean ± SEM, (*n* ≥ 8), *p < 0.05, **p < 0.01, ***p < 0.001.

To further investigate the *in vivo* effects of HY, we established a HFD induced obesity model in ApoE^−/−^ mice (Figure 5A). Keep daily food intake consistent. Visual observation revealed that HFD-fed mice exhibited a significant increase in body size and white adipose tissue (WAT) volume. HY attenuates HFD-induced gain in body weight and WAT volume. (Figure 5B). Throughout the intervention period, mice in the HY treatment group showed a significant decrease in body weight compared with those in the HFD model group, indicating that long-term administration of HY effectively counteracts HFD induced obesity (Figure 5C). Next, major organs were weighed to calculate organ indices. The results showed that HY treatment alleviated organ hypertrophy in ApoE^−^/^−^ mice (Figures 5D,E). These findings suggest that HY exert a regulatory effect on HFD-induced metabolic disorders in mice.

**Figure 6:**
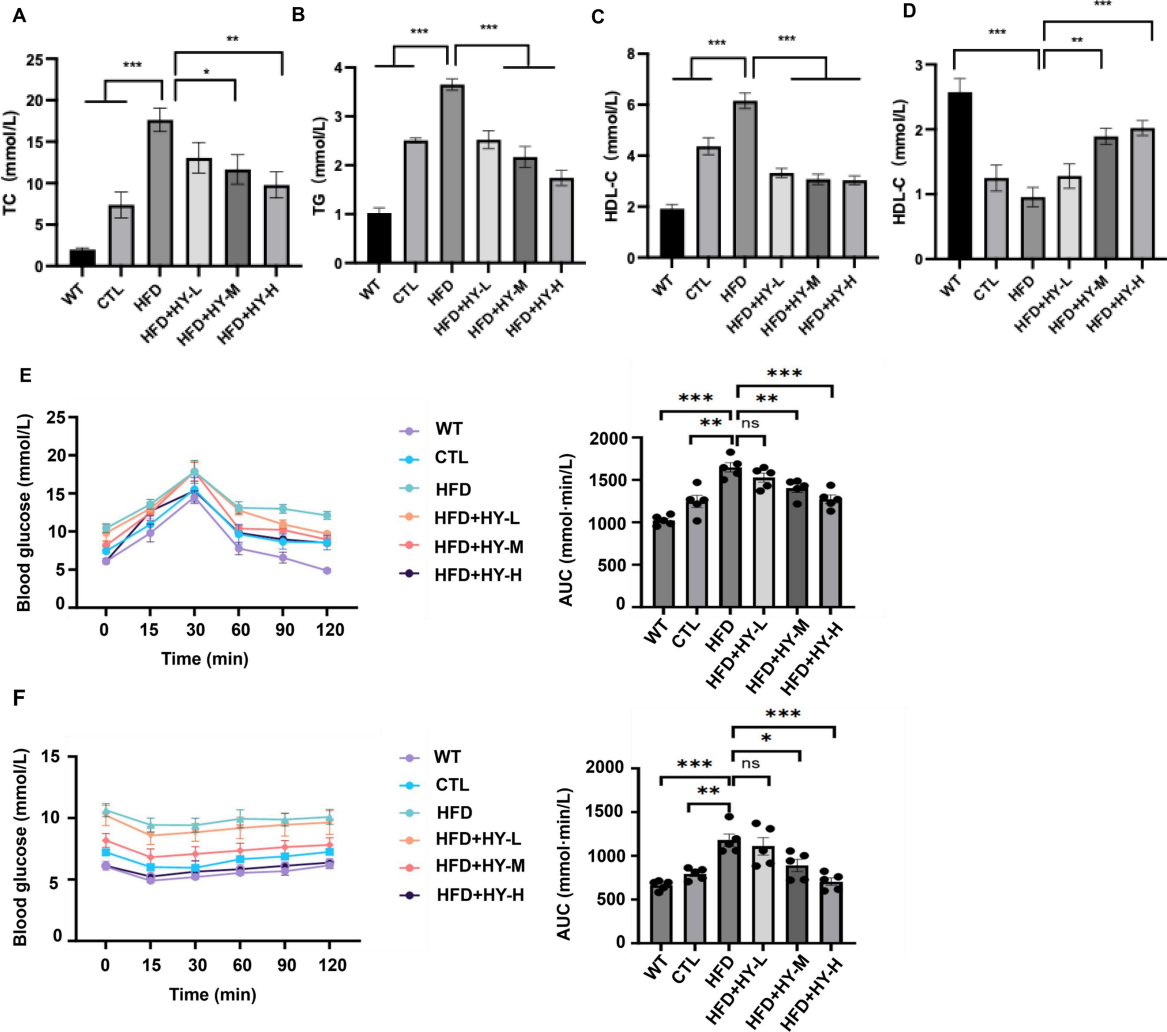
Effects of HY on blood glucose and lipid levels in ApoE^−^/^−^ mice. **(A)** Serum TG levels. **(B)** Serum TC levels. **(C)** Serum HDL-C levels. **(D)** Serum LDL-C levels. **(E)** Blood glucose levels during the oral glucose tolerance test (GTT). AUC calculated from the GTT results. **(F)** Blood glucose levels during the intraperitoneal insulin tolerance test (ITT). AUC calculated from the ITT results. Data are presented as mean ± SEM, (*n* = 5), *p < 0.05, **p < 0.01, ***p < 0.001.

Analysis of serum biochemical parameters showed that compared to WT group, the HFD group developed significant dyslipidemia, characterized by elevated levels of TC, TG, and LDL-C, alongside reduced HDL-C. HY treatment significantly reversed these abnormalities, effectively improving HFD-induced dyslipidemia and demonstrating potential cardiovascular protective benefits (Figures 6A–D). Blood glucose results confirmed that HY also can improve glucose tolerance and enhance insulin sensitivity (Figures 6E,F).

### 3.4 HY attenuated atherosclerotic lesion formation and hepatic steatosis in ApoE^−^/^−^ mice

**Figure 7:**
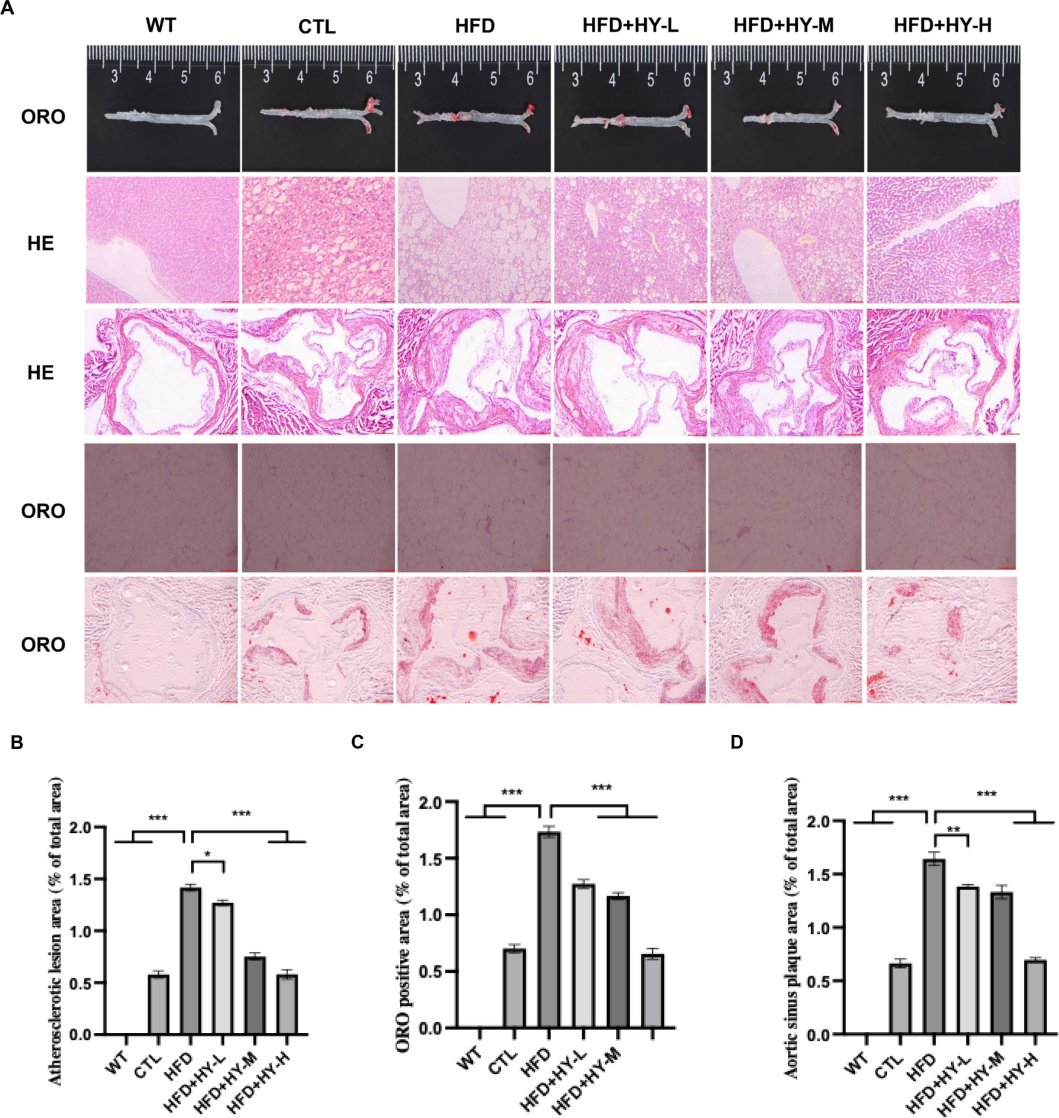
Effects of HY on vascular lesions in ApoE^−^/^−^ mice. **(A)** Representative ORO staining of the entire aorta, H&E staining of liver and aortic sinus sections, as well as ORO staining of aortic sinus and WAT sections. **(B)** Quantification of atherosclerotic plaques in the whole aorta stained with ORO. **(C)** Quantification of lipid accumulation in the aortic root stained with ORO. **(D)** Quantification of atherosclerotic plaques in the aortic root stained with H&E. Data are presented as mean ± SEM, (*n* = 3), *p < 0.05, **p < 0.01, ***p < 0.001.

Histopathological analysis showed that HY significantly reduced lipid deposition in the aorta and aortic sinus. (Figure 7A), with reductions of approximately 45 and 40%, respectively (Figures 7B–D). HFD leads to excessive accumulation of triglycerides in the liver. HY treatment effectively reduced lipid accumulation, thereby improving these morphological abnormalities (Figure 7A). This indicates that HY not only reduced lipid quantity but also contributed to preserving hepatocellular integrity and sinusoid structure. The white fat staining results showed that compared with WT group mice, the model group observed adipocyte hypertrophy and excessive increase in cell volume, indicating the storage of a large amount of TC. Compared with the model group, the mice treated with HY showed significant improvement in the pathological changes mentioned above (Figure 7A). In conclusion, these data finally proved that HY effectively alleviated the development of lipid metabolism disorder and aortic atherosclerosis in obese mice. These findings make HY a potential substance for the prevention or adjuvant treatment of diet induced metabolic disorders and their cardiovascular complications.

### 3.5 HY relieved atherosclerosis by activating GLP-1R/CREB signaling pathway

**Figure 8:**
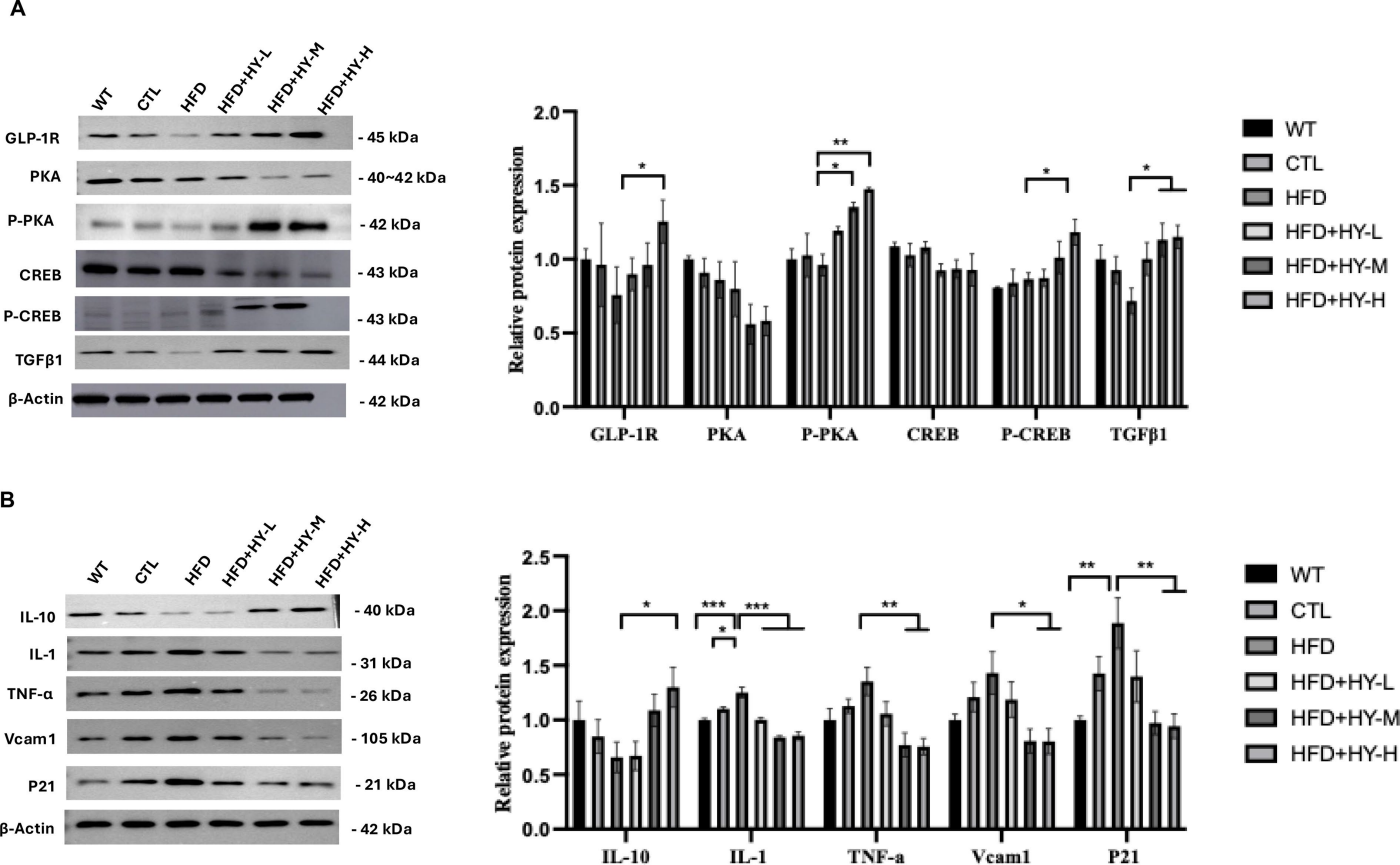
HY regulates the protein expression of the GLP-1R/PKA/CREB. **(A)** Representative bands of various proteins in intestinal tissue and relative protein expression. **(B)** Representative bands of various proteins in cardiovascular tissue and relative protein expression. Data are presented as mean ± SEM, *p < 0.05, **p < 0.01.

Mechanistic studies revealed that HY specifically upregulated the protein expression of GLP-1R and its downstream effector, phosphorylated CREB (p-CREB), in mouse intestinal tissues (Figure 8A). This discovery is crucial as it clearly locates the initial site of action of HY in the intestine, a tissue rich in GLP-1R. The activated p-CREB transport into the nucleus, where it regulates the transcription of a suite of downstream target genes, potentially acting as the molecular switch for initiating a systemic anti-inflammatory program. After activation of the intestinal GLP-1R/cAMP/CREB pathway, the expression and release of factors such as interleukin-10 (IL-10) and transforming growth factor-*β* (TGF-β) were upregulated (Figure 8A). After these factors enter the bloodstream, they synergistically exert anti-inflammatory and anti-aging effects on the aortic wall by inhibiting key pro-inflammatory pathways and promoting plaque stability. In aortic tissue, we observed that HY treatment significantly reduced the protein expression levels of key pro-inflammatory cytokines, including IL-1β, and TNF-*α* (Figure 8B). Meanwhile, the expression of vascular cell adhesion molecule-1 (VCAM-1) and cell aging marker P21 was significantly inhibited (Figure 8B). These observed anti-inflammatory and anti-senescence phenotypes in the aorta spatially correlate with the activation of the GLP-1R/CREB pathway in the gut, suggesting the existence of a systematic signal transmission from the intestine to the cardiovascular system. The results predict a coherent signaling pathway initiated in the gut that systemically modulates cardiovascular inflammation, providing crucial mechanistic insights into the anti-atherosclerotic effects of HY.

## 4 Discussion

Atherosclerotic cardiovascular disease is a leading cause of morbidity and mortality worldwide, and the exploration of its prevention strategies has always been the core topic of medical research (29). Currently, GLP-1RAs have emerged as an important class of therapeutic drugs, given that they have been proven to confer benefits in blood glucose control, weight reduction and cardiovascular health improvement (30, 31). Exemplified by liraglutide, its activation not only regulates metabolism, but has also been proven to have direct or indirect cardiovascular protective effects (32). Such evidence lays a robust theoretical and experimental basis for investigating the therapeutic capacity of the GLP-1R signaling cascade in cardiovascular tissues. Meanwhile, identifying bioactive compounds with novel functions and elucidating their molecular mechanisms from food-medicine homologous materials has garnered increasing research attention for the intervention of hyperlipidemia and associated metabolic syndrome phenotypes (33).

However, peptide-based GLP-1RAs are generally associated with limitations such as the need for injectable administration, relatively high production costs, and potential gastrointestinal side effects (34). Consequently, the development of non-peptide GLP-1RAs, characterized by smaller molecular size, better stability, oral bioavailability, and potentially higher safety, is considered a crucial direction for expanding clinical applications and benefiting a broader patient population (35). Natural active substances provide a vast library of structurally diverse and biologically active compounds, offering advantages for the discovery of novel GLP-1RAs. Previous studies have reported that specific components from natural sources like blueberries and mulberries can interact with the GLP-1R and exhibited beneficial pharmacological effects, highlighting natural products as a highly valuable resource for identifying new GLP-1RAs (13, 36).

Citrus by-products, including pomace, seeds, and peels, are produced in large amounts each year (37). These by-products contain high levels of phenolic compounds and low development costs, and numerous food-derived components can effectively relieve high-fat-induced lipid metabolic disorders (38–40). Given this, the present study focused on HY. Through systematic *in vitro* activity screening and identification, we have characterized HY as a natural substance possessing GLP-1R agonistic activity. We successfully isolated and identified five compounds from the extracts that possess GLP-1R agonistic activity. Subsequent functional validation confirmed that these five compounds act as GLP-1RAs. To comprehensively evaluate the anti-atherosclerotic efficacy of HY, we established an ApoE^−^/^−^ mouse model of AS and administered HY via long-term oral gavage. *In vivo* data clearly demonstrated that HY treatment significantly reduced atherosclerotic plaque formation in the aorta. Meanwhile, this intervention conferred systemic metabolic benefits, including body weight reduction, improved lipid metabolism, and enhanced glucose tolerance.

Mounting evidence indicates that gut microbiota dysbiosis triggers abnormal immune activation and overproduction of pro-inflammatory cytokines (41, 42). These circulating pro-inflammatory mediators migrate to cardiovascular tissues, aggravating vascular inflammation and accelerating the initiation of cardiovascular complications. Such immune crosstalk directly connects intestinal homeostasis with cardiovascular pathological lesions, underscoring the critical role of immune-mediated gut-cardiac axis signaling (43).

In the present study, oral Huyou peel extract (HY) firstly activates GLP-1R on intestinal epithelial cells. This upstream stimulus initiates the intracellular cyclic adenosine monophosphate (cAMP) signaling pathway, which further drives the phosphorylation of protein kinase A (PKA), and the generated phosphorylated PKA (p-PKA) subsequently triggers the phosphorylation and activation of transcription factor cAMP response element-binding protein (CREB). Activated CREB modulates the transcription of downstream target genes to stimulate the synthesis and secretion of systemic anti-inflammatory signaling mediators. These gut-derived protective signals are delivered to distal vascular tissues through the circulatory system (44). At the site of the aortic wall, they exert local effects, specifically characterized by the effective suppression of vascular inflammatory responses and the downregulation of key proteins associated with cellular senescence. This long-distance signal transmission ultimately contributes collectively to the mitigation and slowing of the atherosclerotic pathological process.

Prolonged oxidative stress induced by lipid overload disrupts transcriptional networks that maintain vascular homeostasis. Cross-species comparative research has confirmed that the cAMP/PKA/CREB cascade acts as an evolutionarily conserved core signaling hub coordinating vertebrate tissue stress responses; excess reactive oxygen species (ROS) disrupt the phosphorylation equilibrium of PKA and repress CREB-mediated anti-inflammatory transcription, thereby accelerating atherosclerotic deterioration (45). Consistent with this mechanistic paradigm, our data demonstrate that flavonoid components in HY rescue impaired p-PKA/p-CREB signaling under hyperlipidemic stress, which alleviates aortic inflammation and cellular senescence. Apart from this canonical protein signaling axis, food-derived bioactive agents such as HY may also interfere with upstream genetic and epigenetic regulators, including the type 2 diabetes-associated MTHFR single-nucleotide polymorphism (rs1801133) that modulates target mRNA abundance (46) and interconnected microRNA-lncRNA regulatory networks recognized as promising therapeutic targets (47). Further investigation into the crosstalk between HY and these upstream genetic/epigenetic regulators will deepen our mechanistic understanding of how HY modulates downstream vascular gene expression and ameliorates metabolic syndrome manifestations.

Despite the promising anti-atherosclerotic efficacy and systemic metabolic benefits demonstrated by HY in this study, several limitations must be acknowledged. Although our phytochemical identification verified that HY extract mainly consists of citrus flavonoids without detectable p-synephrine or other toxic citrus alkaloids, comprehensive standardized toxicological data supporting its long-term oral safety remain insufficient in the existing published literature. At present, there is a lack of systematic toxicological assessments for HY extract, including acute toxicity, repeated-dose toxicity, genotoxicity, reproductive toxicity, carcinogenicity tests, as well as LD₅₀ measurement and NOAEL determination. Although repeated oral administration of flavonoid-rich extracts from Citrus Changshan Huyou in rodent models has not induced obvious toxic phenotypes in existing preliminary studies, complete toxicological characterization is still required to fully guarantee its safety as a functional food raw material. Therefore, follow-up research will focus on completing a full set of toxicological experiments to systematically evaluate the oral safety of HY extract, which can further fill the research gap and provide more sufficient evidence for its subsequent food development and clinical transformation.

Beyond gut-heart axis-mediated systemic inflammation and macrophage infiltration, severe metabolic disturbance in ApoE^−^/^−^ mice establishes a permissive pathological microenvironment that aggravates macrovascular injury and coronary structural lesions. Chronic hyperglycemia triggers excessive deposition of advanced glycation end-products (AGEs), which act as vital molecular mediators connecting metabolic abnormalities to coronary artery disease (CAD). AGEs form irreversible cross-links with extracellular matrix proteins, impairing vascular elasticity and accelerating arterial stiffness. Clinical data strongly support this pathological correlation: elevated circulating Nε-carboxymethyl-lysine (CML, a canonical AGE biomarker) correlates positively with increased inflammatory biomarkers and aggravated coronary plaque burden in diabetic CAD patients (48). Meanwhile, sustained activation of the AGE-RAGE signaling axis exacerbates endothelial dysfunction and induces pathological phenotypic switching of vascular smooth muscle cells (VSMCs). Such cellular transformation drives vascular fibrotic remodeling and luminal narrowing, leading to progressive macrovascular stenosis, enhanced plaque vulnerability, and a higher risk of acute coronary syndromes. By modulating GLP-1R signaling, HY alleviates upstream inflammatory responses through the gut-heart axis and simultaneously suppresses hyperglycemia-induced AGE overproduction and subsequent AGE-RAGE pathogenic signaling. The ability of HY to attenuate macrovascular stenosis and coronary fibrotic remodeling underscores its therapeutic potential to block the progression from mild metabolic dysfunction to severe obstructive cardiovascular complications.

## 5 Conclusion

In conclusion, this study demonstrates that HY represents a novel food-derived source of natural GLP-1RAs, exhibiting favorable anti-atherosclerotic efficacy and metabolic safety in animal models. Importantly, HY and its active components offer distinct advantages associated with food-derived GLP-1RAs, including oral applicability, low toxicity, and potential for cost-effective production, supporting their development as functional food ingredients. Our work not only provides a promising candidate substance and scientific foundation for developing functional foods targeting atherosclerosis prevention but also offers an innovative theoretical framework for designing cardiovascular protective strategies based on citrus flavanones and other natural products.

## Data Availability

The original contributions presented in the study are included in the article/Supplementary material, further inquiries can be directed to the corresponding authors.

## Footnotes

1
https://pubchem.ncbi.nlm.nih.gov

2
https://www.rcsb.org
